# Multimodal Therapeutic Exercises and Their Role in Mitigating Chemotherapy-Induced Peripheral Neuropathy: A Longitudinal Study on Patient Outcomes

**DOI:** 10.7759/cureus.74214

**Published:** 2024-11-22

**Authors:** Trupti Yadav, G. Varadharajulu, Rashmi Gudur

**Affiliations:** 1 Department of Oncologic Physiotherapy, Krishna College of Physiotherapy, Krishna Vishwa Vidyapeeth (Deemed to be University), Karad, IND; 2 Department of Neurosciences, Krishna College of Physiotherapy, Krishna Vishwa Vidyapeeth (Deemed to be University), Karad, IND; 3 Department of Oncology, Krishna Vishwa Vidyapeeth (Deemed to be University), Karad, IND

**Keywords:** carboplatin, chemotherapy-induced peripheral neuropathy, iadl (instrumental activities of daily living), multimodal therapeutic exercise, paclitaxel

## Abstract

Introduction

A common side effect post chemotherapy is chemotherapy-induced peripheral neuropathy (CIPN). The purpose of this study was to investigate the effect of a multimodal exercise program compared to standard physical therapy in treating CIPN symptoms and improving daily living skills.

Aim

The aim of this study was to evaluate the effect of multimodal therapeutic exercises and their role in mitigating CIPN symptoms on the neuropathy score and instrumental activities of daily living.

Methodology

Seventy-eight participants were divided into two groups: multimodal exercise program (Group A) and standard physical therapy (Group B) who fulfilled the inclusion criteria of age 25-55 years with CIPN (grade 2 or 3). Pre- and post-assessment was done using the modified Total Neuropathy Score (mTNS) and Lawton Instrumental Activities of Daily Living Scale (L-IADL).

Results

A significant improvement in the mTNS was seen in Group A (14.68 ± 1.66 to 7.03 ± 1.49, p<0.0001) compared to Group B (14.98 ± 1.54 to 13.66 ± 1.34, p=0.00116). L-IADL scores also showed more improvement in Group A (7.54 to 11.23, p<0.0001) versus Group B (5.51 to 7.35, p=0.001). A statistically significant improvement was seen (p<0.0001) for both outcome measures post-intervention.

Conclusion

A superior efficacy was demonstrated by the multimodal exercise program demonstrated in reducing CIPN symptoms and improving functional outcomes compared to standard physical therapy. These findings suggest that comprehensive, multimodal exercise interventions may be more effective in managing CIPN and enhancing quality of life for cancer patients undergoing neurotoxic chemotherapy.

## Introduction

Cancer is a leading cause of death globally, characterized by the rapid proliferation of abnormal cells that invade surrounding tissues is cancer [1]. An increase in cancer cases and deaths between 2020 and 2022 was seen in India on recent data from the ICMR National Cancer Registry Programme. A variety of therapeutic approaches encompasses cancer treatment [2]. The initial intervention in the treatment approach is surgery followed by post-surgical care, which often involves additional treatments such as hormone therapy, chemotherapy, and radiation therapy [3].

A crucial role in the treatment of cancer is played by chemotherapy which is administered in various ways. Chemotherapy may be given in the form of adjuvant therapy (after primary treatment) or neoadjuvant therapy (before primary treatment). The purpose of chemotherapy is to shrink tumors, slow cancer progression, and improve overall patient prognosis [3,4].

A wide range of chemotherapy regimes are available for healthcare providers. The selection of drugs and their combination is a nuanced process, tailored to each patient's unique circumstances. It depends on several factors like the type of cancer, stage of the disease, cancer's aggressiveness, patient's overall health status, and intended treatment strategy. This personalized approach ensures that each patient receives the most appropriate and effective chemotherapy regimen for their particular case [3,4].

Chemotherapeutic agents that cause neurotoxic effects on the peripheral nervous system are commonly used to treat prevalent cancer types. The six primary classes of these chemotherapeutic agents that lead to symptoms of chemotherapy-induced peripheral neuropathy (CIPN) like peripheral sensory, motor, and autonomic neuron damage include platinum-based antineoplastics (notably oxaliplatin and cisplatin), vinca alkaloids (such as vincristine and vinblastine), epothilones (like ixabepilone), taxanes (including paclitaxel and docetaxel), proteasome inhibitors (such as bortezomib), and immunomodulatory drugs (like thalidomide). Out of these, the most neurotoxic agents that cause CIPN are typically platinum-based drugs, taxanes, ixabepilone, and thalidomide and its derivatives [5].

The unique mechanism of action of paclitaxel, a member of the taxane class of chemotherapeutic agents, has garnered significant attention in cancer treatment as this compound thereby inhibits cell division in both cancerous and non-cancerous proliferating cells due to disruption of mitosis by interfering with microtubule function. A substantial proportion of patients manifest as motor impairment due to paclitaxel-induced neuropathy. The prevalence of this side effect ranges from 10% to over 60%, with variability attributed to factors such as the treatment regimen, patient age, and comorbidities [6].

A complex interplay of various physiological mechanisms results in paclitaxel-induced motor dysfunction. An inflammatory cascade in muscle tissue which activates immune cells and promotes the release of cytokines and other inflammatory mediators is initiated by paclitaxel [7]. This causes sensitization of nerve endings, exacerbating neuropathic pain. Concurrently, paclitaxel induces oxidative stress in muscle cells by elevating reactive oxygen species (ROS) production while compromising antioxidant defenses. Inflammatory processes may be further exacerbated by the oxidative damage to cellular components [7,8].

Moreover, paclitaxel interferes with microtubule dynamics, which are essential for proper sarcomere organization and function which in turn disrupts mitochondrial function in muscle cells. This in turn leads to structural and functional abnormalities in sarcomeres, contributing to motor impairment. Also, modulation of the activity of calcium, sodium, and potassium channels in various cell types, including muscle cells, is seen. The generation of action potentials necessary for muscle contraction is compromised due to alterations in ion channel function, which can perturb normal calcium dynamics, membrane potential, and cellular excitability [9,10].

The alterations in intracellular signaling pathways and cytoskeletal organization are due to the drug's impact on microtubule dynamics which indirectly affects the organization and function of actin and myosin filaments within muscle cells. This leads to impairment in ATP synthesis, reducing energy availability in muscle cells and predisposing them to dysfunction. As a result, patients may experience a range of symptoms including pain, muscle cramps, fatigue, and weakness, further significantly impairing fine motor skills and interfering with activities of daily living (ADLs), such as object manipulation, mobility, and self-care tasks, and ultimately resulting in substantial motor dysfunction [11,12].

Carboplatin, a platinum-based drug, activates glial cells, which subsequently attract and activate immune cells, leading to the release and elevation of pro-inflammatory cytokines (such as interleukins and chemokines). As a result, this process leads to nociceptor sensitization and hyperexcitability of peripheral neurons and, together with ROS, contributes to blood-brain barrier damage. These events culminate in the development of neuroinflammation. The enzyme, protein, and lipid damage within neurons, as well as dysregulation of calcium homeostasis, which triggers apoptotic changes in peripheral nerves and dorsal root ganglion (DRG) cells, occurs due to increased ROS production as a result of mitochondrial damage by platinum-based drugs. The activity of Na+, K+, and transient receptor potential ion channels, causing the hyperexcitability of peripheral neurons is affected by platinum-based drugs. These processes collectively have the potential to alter the excitability of peripheral neurons. The primary mechanism responsible for the neurotoxicity associated with this class of chemotherapeutics is accumulation of platinum adducts in DRG and trigeminal ganglion neurons. This phenomenon proportionally relates to the severity of neuropathic behavior in laboratory animals. The exact mechanism of platinum-based agent neurotoxicity in humans remains a topic of discussion; various experimental and preclinical studies have explained the processes likely involved in the pathogenesis of neuropathy [5].

Various treatment approaches, both pharmacological and non-pharmacological ones, are used in the treatment of CIPN symptoms and in improving patients' functional capacity. Nonpharmacological treatments like exercise-based interventions have proved to have beneficial effects [13]. One form of such an exercise regime is multimodal therapeutic exercise programs, which combine various forms of physical activity and targeted exercises and have shown potential in addressing multiple aspects of CIPN and its associated functional limitations [14].

The present study focuses on analyzing the influence of a comprehensive multimodal therapeutic exercise program on CIPN and ADL skills in patients on chemotherapy treatment with paclitaxel and carboplatin [15]. This study evaluates the effectiveness of a multimodal exercise intervention in reducing neuropathic symptoms thereby enhancing sensorimotor function and improving patients' ability to perform ADLs.

The multimodal therapeutic exercise program focuses on aerobic training, resistance exercises, balance and proprioception activities, and specific neuromuscular exercises tailored to address CIPN-related impairments [16]. This is based on the concept of targeting multiple physiological systems, counteracting the neurotoxic effects of chemotherapy, and promoting neuroplasticity, thus maintaining or improving overall physical function.

This research tries to minimize a critical gap in CIPN management and explores a potentially cost-effective, non-invasive intervention that could be integrated into standard oncology care [17]. The findings of this study may contribute to the development of evidence-based guidelines for CIPN prevention and management, ultimately enhancing the quality of life and treatment outcomes for cancer patients receiving neurotoxic chemotherapy.

## Materials and methods

Study design

This longitudinal study employed an eight-week protocol to evaluate the efficacy of a multimodal exercise program on CIPN in patients receiving treatment with carboplatin and paclitaxel.

Participants

A total of 78 participants fulfilling the inclusion criteria of age 25 to 55 years, undergoing or having completed chemotherapy with carboplatin and paclitaxel irrespective of the type of cancer, and who presented with CIPN graded as 2 or 3 according to the Common Terminology Criteria for Adverse Events (CTCAE) were recruited from oncology clinics.

Assessment tools

Prior to the initiation of the intervention and after completion of intervention, baseline assessments were conducted using the Total Neuropathy Score (TNS) to evaluate the severity of neuropathy symptoms and the Lawton-Brody Instrumental Activities of Daily Living Scale (IADL) to assess functional dependence in daily living activities. 

Interventions

The interventional group, Group A, underwent a structured multimodal therapeutic exercise program which included chair aerobic exercises ( diagonal toe touch, criss-cross (along with arm movement), marching with arm movement, alternate hand, and leg movements, punches, claps on head, etc.), static and dynamic balance training using stable and unstable surface and resistance training for upper and lower limb, while the control group received standard physical therapy aimed at maintaining mobility and managing symptoms. The progression of exercises in the interventional group was done every four weeks which included chair aerobics increase in five sets with progressive increase in speed, 10% increase in one repetition maximum, and progress from static balance training to dynamic balance training. The treatment was given for eight weeks, four times weekly. 

Outcome measures

Post-assessment after eight weeks severity and functional dependence was assessed using TNS and IADL. The effects of the therapies on CIPN symptoms and functional independence were assessed using statistical analyses, which included between-group comparisons and effect size assessments.

Ethical considerations

The ethics committee of Krishna Vishva Vidyapeeth approved the study protocol (496/2022-23). Informed consent was obtained from the subjects before enrollment.

## Results

Table 1 depicts the age distribution which shows that most participants fall within the 36-45 age group, followed by the 46-55 and 25-35 age groups. The above data show that there is an even distribution of participants in each age group signifying a relatively balanced age representation across the study, with no significant lean toward any particular age range.

**Table 1:** Baseline characteristics of patients in both groups

Age	Group A	Group B	Total	Percentage
25-35	15	9	24	30.7
36-45	12	16	28	35.8
46-55	12	14	26	33.3

The data presented in Table 2 summarize the results of the pre- and post-intervention assessments of the mTNS for two groups involved in the study: Group A (multimodal exercise program) and Group B (control group receiving standard physical therapy).

**Table 2:** Results of the pre- and post-intervention assessments of the mTNS for Group A and Group B mTNS: Modified Total Neuropathy Score

	Pre	Post	t-value	p-value
Group A	14.68 ±1.66	7.03 ±1.49	32.84	<0.0001
Group B	14.98 ±1.54	13.66 ±1.34	3.37	0.00116
	0.895	<0.0001		

In Table 2, Group A exhibited a significant reduction in neuropathy symptoms, with 7.65 as the mean difference in pre and post-intervention scores. The pre- and post-evaluation indicates a statistically significant difference with a p-value of <0.0001 demonstrating a highly effective impact of the multimodal exercise program on alleviating CIPN symptoms.

Group B, in contrast, showed a smaller reduction in neuropathy symptoms, from a mean difference of 1.32 pre- and post-intervention scores. The p-value of 0.00116 suggests that while the change is also statistically significant, the magnitude of improvement is markedly less than that observed in Group A.

Overall, the analysis indicates that the multimodal exercise program (Group A) resulted in a significantly greater reduction in CIPN symptoms compared to the standard physical therapy regimen (Group B). The p-values for both groups underscore the robustness of the findings, with Group A achieving a level of significance that reflects a substantial clinical benefit. This suggests that multimodal exercise may be a more effective intervention for managing CIPN in patients undergoing chemotherapy with carboplatin and paclitaxel.

In Table 3, in Group A, the mean difference of 3.69 pre- and post-intervention scores exhibited a significant improvement in L-IADL. The p-value of <0.0001 indicates that this change is statistically significant, demonstrating a highly effective impact of the intervention on improving instrumental activities of daily living in this group.

**Table 3:** Results of the pre- and post-intervention assessments of the L-IADL score for Group A and Group B L-IADL: Lawton Instrumental Activities of Daily Living Scale

	Pre	Post	t-value	p-value
Group A	7.54 ±1.46	11.23 ±2.24	14.08	<0.0001
Group B	5.51±1.59	7.35±2.36	6.84	0.001
	0.032	<0.0001		

In Group B, in contrast, the mean difference of 1.84 pre- and post-intervention scores showed a smaller improvement in L-IADL scores. The p-value of 0.001 shows a statistically significant change, but less remarkable than that observed in Group A.

From the above observations, it can be interpreted that the intervention administered to Group A resulted in a significantly greater improvement in post-scores of L-IADL as compared to Group B. The between-group p-values further underscore that, with a pre-intervention p-value of 0.032 there is a baseline difference which shows homogeneity in the pre-intervention values, and a post-intervention p-value of <0.0001 highlighting the substantial differential impact between the two groups. These results suggest that the intervention in Group A is more effective in enhancing IADL.

## Discussion

The current study focused on the efficacy of a multimodal exercise compared to standard physical therapy in treating CIPN by carboplatin and paclitaxel. The results of the study demonstrate significant improvements in both the outcome measures i.e. reduction in neuropathy symptoms and improvement in the IADL in the patients who were in the experimental group and received multimodal exercises.

In consideration of the baseline characteristic of age, a balanced distribution of age distribution was observed across three age groups: 25-35 years (30.7%), 36-45 years (35.8%), and 46-55 years (33.3%). This gives a generalizability of the findings, suggesting that the observed benefits of the interventional group with a multimodal exercise program may be applicable across a broad spectrum of age in adult cancer patients experiencing CIPN symptoms. This even distribution minimizes the risk of bias in the study outcomes and equal age distribution is important for understanding the efficacy of treatments across different life stages.

The first outcome measure used was the TNS which showed a remarkable improvement in the scores of Group A where the multimodal exercise was given, a substantial reduction in neuropathy symptoms, with mean mTNS scores decreasing from 14.68 to 7.03 (p<0.0001). This significant improvement suggests that the multimodal approach effectively reduces the severity of CIPN symptoms.

In contrast, Group B, which received routine physical therapy, showed a mild improvement, with mean mTNS scores decreasing from 14.98 to 13.66 (p=0.00116). Although the results are statistically significant, the amount of improvement proves less as compared to the multimodal exercise group.

The between-group comparison of both the groups showed that a remarkable improvement was observed in the multimodal exercise group (p<0.0001). The findings might be so because the multimodal exercise focuses on multiple systems rather than focusing on one component thus proving to be effective [18].

Streckmann et al., in their systematic review of CIPN, reported that studies focusing on multiple forms of exercise had better outcomes compared to sticking to just one form of exercise [16]. The present study also focused on elements such as aerobic exercise, resistance training, and balance exercises, which appears to corroborate their conclusions. A similar result was found in the study performed by Zimmer et al. found a mean reduction of 2.5 points in mTNS scores after a 12-week exercise intervention, whereas the present study showed a mean reduction of 7.65 points [14].

The second outcome measure used was L-IADL scores. Group A L-IADL scores showed a remarkable improvement in scores, increasing from 7.54 to 11.23 (p<0.0001) while Group B showed an increase from 5.51 to 7.35 (p=0.001). The between-group comparison post-intervention showed p<0.0001 thus proving the superior efficacy of the multimodal exercise program in enhancing functional outcomes.

The result of the present study correlates with the study of Zimmer et al. who proved that a multimodal therapeutic exercise had a significant role in the improvement of balance, strength, and QOL; thus an overall improvement in these outcomes might also have resulted in improvement in the activities of daily living [14]. The present study focuses on the protocol which focuses on aerobic training, strength training, and balance training; thus due to a multimodal approach, there is reduction in the symptoms of CIPN which in turn might have an improvement in the IADL. Another factor might be the focus on strength training which improved the strength of muscles thus helping in improving the instrumental activities of daily living.

In future studies, a long-term follow-up can be made so that the long-term effect can be monitored and another group of chemotherapy drugs can be included.

## Conclusions

Thus, from the findings of the present study, it can be concluded that the multimodal therapeutic exercise program helps to reduce the symptoms of CIPN and improve the IADL. However, the multimodal therapeutic exercise program which focuses on a multidimensional treatment approach of aerobic exercises, resistance training, and balance training helps to reduce the symptoms of CIPN and improve the IADL more effectively.
